# Supplementary material for: Automated clear cell renal carcinoma grade classification with prognostic significance
Source: PLoS One. 2019 Oct 3;14(10):e0222641. doi: 10.1371/journal.pone.0222641 (PMC6776313; doi:10.1371/journal.pone.0222641)
Supplement: S1 Table — These methods were implemented using the glmnet and caret packages in R. (DOCX) [file pone.0222641.s001.docx]

**S1 Table. The average area under the receiver-operator characteristic curves (AUC ROC) for each machine learning method using the training set after 100 iterations of random 10% hold out.** These methods were implemented using the glmnet and caret packages in R.

| Rank | Method | Optimal hyper parameter | AUC ROC using the training set | Standard Deviation of the AUC ROC |
| --- | --- | --- | --- | --- |
| 1 | Ensemble | - | 0.839 | - |
| 2 | Lasso | λ = 0.0101 | 0.834 | 0.082 |
| 3 | Elastic net | λ = 0.0101 | 0.823 | 0.085 |
| 4 | Ridge | λ = 0.0201 | 0.809 | 0.087 |
| 5 | Neural network | Weight decay = 0.1; hidden units = 6 | 0.800 | 0.090 |
| 6 | Linear support vector machine | Cost = 0.5 | 0.799 | 0.088 |
| 7 | Random forest | Number of trees = 1500, Randomly selected predictors = 9 | 0.781 | 0.091 |
