# Supplementary material for: Automated clear cell renal carcinoma grade classification with prognostic significance
Source: PLoS One. 2019 Oct 3;14(10):e0222641. doi: 10.1371/journal.pone.0222641 (PMC6776313; doi:10.1371/journal.pone.0222641)
Supplement: S2 Table — (DOCX) [file pone.0222641.s002.docx]

**S2 Table.** The association of the nine nuclear morphological features with Fuhrman’s grade in the 277 concordant cases (using the 2-tiered grading system).

|  | **Four-tiered Grading** | | **Two-tiered Grading** | |
| --- | --- | --- | --- | --- |
|  | **Median** | **MAD** | **Median** | **MAD** |
| **p-values** |  |  |  |  |
| Area | 9.20E-02 | **7.86E-04** | **3.36E-02** | **1.69E-04** |
| Roundness | **9.17E-06** | **6.43E-03** | **1.18E-05** | **6.63E-04** |
| Elongation | **5.78E-07** | **6.54E-06** | **1.30E-05** | **1.05E-02** |
| Flatness | **5.78E-07** | **6.54E-06** | **1.30E-05** | **1.05E-02** |
| Perimeter | **9.11E-06** | **8.10E-06** | **6.31E-06** | **1.08E-05** |
| Equivalent Spherical Perimeter | 9.20E-02 | **1.40E-04** | **3.36E-02** | **2.92E-05** |
| Equivalent Spherical Radius | 9.20E-02 | **1.40E-04** | **3.36E-02** | **2.92E-05** |
| Minor Axis of the Ellipse Fit | 7.21E-01 | **1.63E-04** | 3.87E-01 | **3.62E-05** |
| Major Axis of the Ellipse Fit | **1.90E-04** | **6.19E-06** | **8.04E-05** | **5.03E-05** |
| **FDR adjusted values** |  |  |  |  |
| Area | 1.03E-01 | **8.84E-04** | **3.78E-02** | **2.53E-04** |
| Roundness | **2.06E-05** | **6.43E-03** | **2.92E-05** | **8.52E-04** |
| Elongation | **2.60E-06** | **1.82E-05** | **2.92E-05** | **1.05E-02** |
| Flatness | **2.60E-06** | **1.82E-05** | **2.92E-05** | **1.05E-02** |
| Perimeter | **2.06E-05** | **1.82E-05** | **2.92E-05** | **8.15E-05** |
| Equivalent Spherical Perimeter | 1.03E-01 | **2.10E-04** | **3.78E-02** | **8.15E-05** |
| Equivalent Spherical Radius | 1.03E-01 | **2.10E-04** | **3.78E-02** | **8.15E-05** |
| Minor Axis of the Ellipse Fit | 7.21E-01 | **2.10E-04** | 3.87E-01 | **8.15E-05** |
| Major Axis of the Ellipse Fit | **3.42E-04** | **1.82E-05** | **1.45E-04** | **9.05E-05** |

Median absolute deviation, MAD; red text indicates a value that is p<0.05 or false discovery rate (fdr) <0.05.
