# Supplementary material for: Automated clear cell renal carcinoma grade classification with prognostic significance
Source: PLoS One. 2019 Oct 3;14(10):e0222641. doi: 10.1371/journal.pone.0222641 (PMC6776313; doi:10.1371/journal.pone.0222641)
Supplement: S3 Table — (DOCX) [file pone.0222641.s003.docx]

**S3 Table. Contingency tables of Fuhrman’s grade between TCGA and Pathologist 1 with Pathologist 2 among the 118 discordant cases.**

|  | 4-tiered grading system | | | |  | 2-tiered grading system | |
| --- | --- | --- | --- | --- | --- | --- | --- |
|  | Pathologist 2 | | | |  | Pathologist 2 | |
|  | Grade 1 | Grade 2 | Grade 3 | Grade 4 |  | Low Grade | High Grade |
| TCGA |  |  |  |  | TCGA |  |  |
| Grade 1 | 0 | 0 | 0 | 0 | Low Grade | 9 | 19 |
| Grade 2 | 0 | 9 | 16 | 3 | High Grade | 53 | 37 |
| Grade 3 | 2 | 46 | 29 | 1 |  |  |  |
| Grade 4 | 0 | 5 | 6 | 1 |  |  |  |
| Pathologist 1 |  |  |  |  | Pathologist 1 |  |  |
| Grade 1 | 2 | 11 | 3 | 0 | Low Grade | 53 | 37 |
| Grade 2 | 0 | 40 | 32 | 2 | High Grade | 9 | 19 |
| Grade 3 | 0 | 9 | 15 | 3 |  |  |  |
| Grade 4 | 0 | 0 | 1 | 0 |  |  |  |
