# Supplementary material for: Automated clear cell renal carcinoma grade classification with prognostic significance
Source: PLoS One. 2019 Oct 3;14(10):e0222641. doi: 10.1371/journal.pone.0222641 (PMC6776313; doi:10.1371/journal.pone.0222641)
Supplement: S4 Table — (DOCX) [file pone.0222641.s004.docx]

**S4 Table. The association of re-assigned grades for discordant cases.**

|  | **Re-assigned Consensus Manual Grade** | | |
| --- | --- | --- | --- |
|  | **Hazard**  **Ratio** | **(95% CI)** | ***p*-value** |
| **A.** **Most frequent grade among TCGA, Pathologist 1, and Pathologist 2** | | | |
| Model A: Crude | 1.09 | (0.63, 1.89) | 0.75 |
| Model B: Adjusted for Age and Gender | 1.21 | (0.70, 2.10) | 0.50 |
| Model C: Adjusted for Age, Gender, and Stage | 1.15 | (0.66, 2.00) | 0.62 |
| **B.** **Most frequent grade among Pathologist 1, Pathologist 2, and Computer** | | | |
| Model A: Crude | 1.33 | (0.77, 2.29) | 0.31 |
| Model B: Adjusted for Age and Gender | 1.42 | (0.81, 2.47) | 0.22 |
| Model C: Adjusted for Age, Gender, and Stage | 1.24 | (0.70, 2.20) | 0.46 |

Confidence Interval, CI
