# Supplementary figures and images for: Automated clear cell renal carcinoma grade classification with prognostic significance
Source: PLoS One. 2019 Oct 3;14(10):e0222641. doi: 10.1371/journal.pone.0222641 (PMC6776313; doi:10.1371/journal.pone.0222641)

A. Furhman Grading (4–tiered) of the 395 TCGA Cases

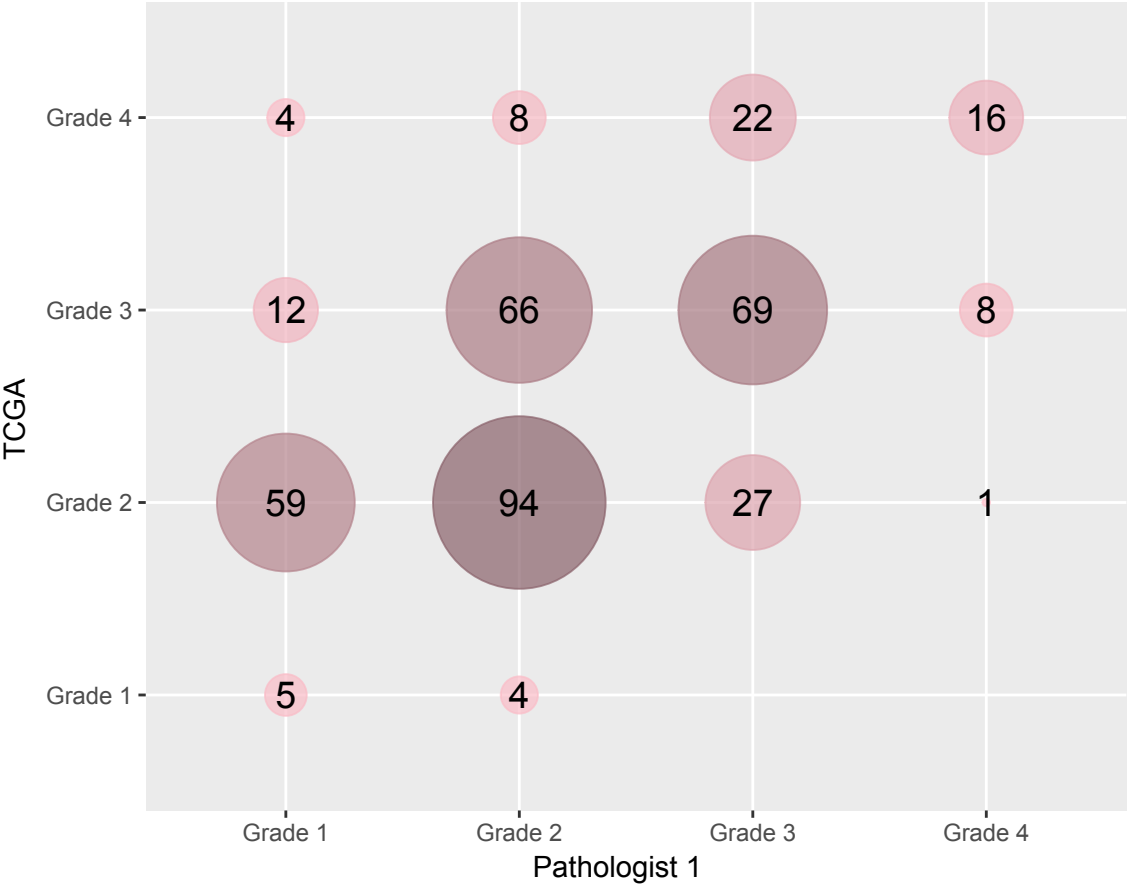

B. Furhman Grading (2–tiered) of the 395 TCGA Cases

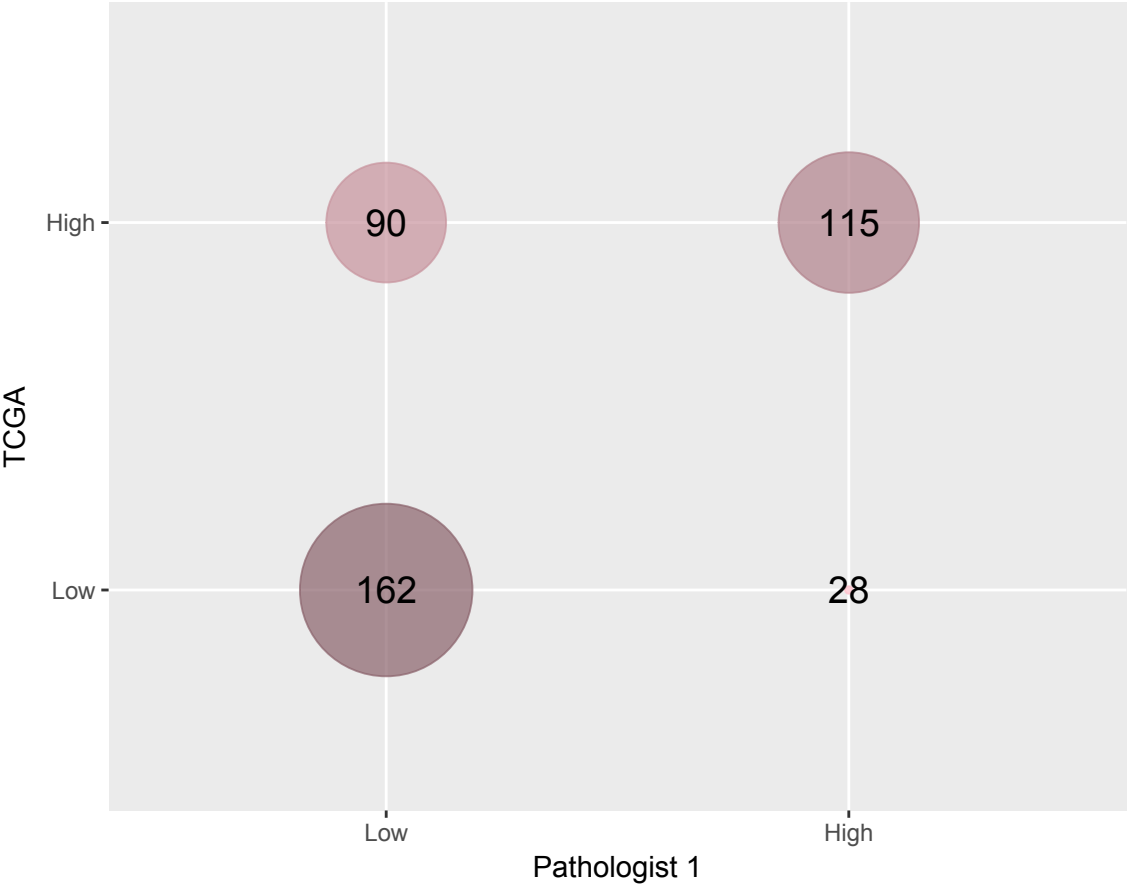

Supplement: S1 Fig — (A) The agreement between the TCGA and Pathologist 1 using the 4-tiered grading was poor (frequency of agreement = 0.47, Cohen’s kappa = 0.20). (B) The agreement improved to moderate when using the 2-tiered grading system (frequency of agreement = 0.70, Cohen’s kappa = 0.41). (PDF) [file pone.0222641.s005.pdf]
