# Supplementary material for: Automated clear cell renal carcinoma grade classification with prognostic significance
Source: PLoS One. 2019 Oct 3;14(10):e0222641. doi: 10.1371/journal.pone.0222641 (PMC6776313; doi:10.1371/journal.pone.0222641)

S2 Fig. These plots contain the 277 cases that were concordant between TCGA and Pathologist 1 using the 2-tiered grading system. Nine morphological features (**1:** Area, 2: Roundness, **3:** Elongation, **4:** Flatness, **5:** Perimeter, **6:** Equivalent Spherical Perimeter, **7:** Equivalent Spherical Radius, **8:** Minor Axis of the Ellipse Fit, and **9:** Nuclei Major Axis of the Ellipse Fit) were plotted with their median and median absolute deviation values. Each feature and measure was stratified either by the 4-tiered (**A and B**) or 2-tiered (**C and D**) grades assigned by Pathologist 1, respectively.

1 Nuclei Area

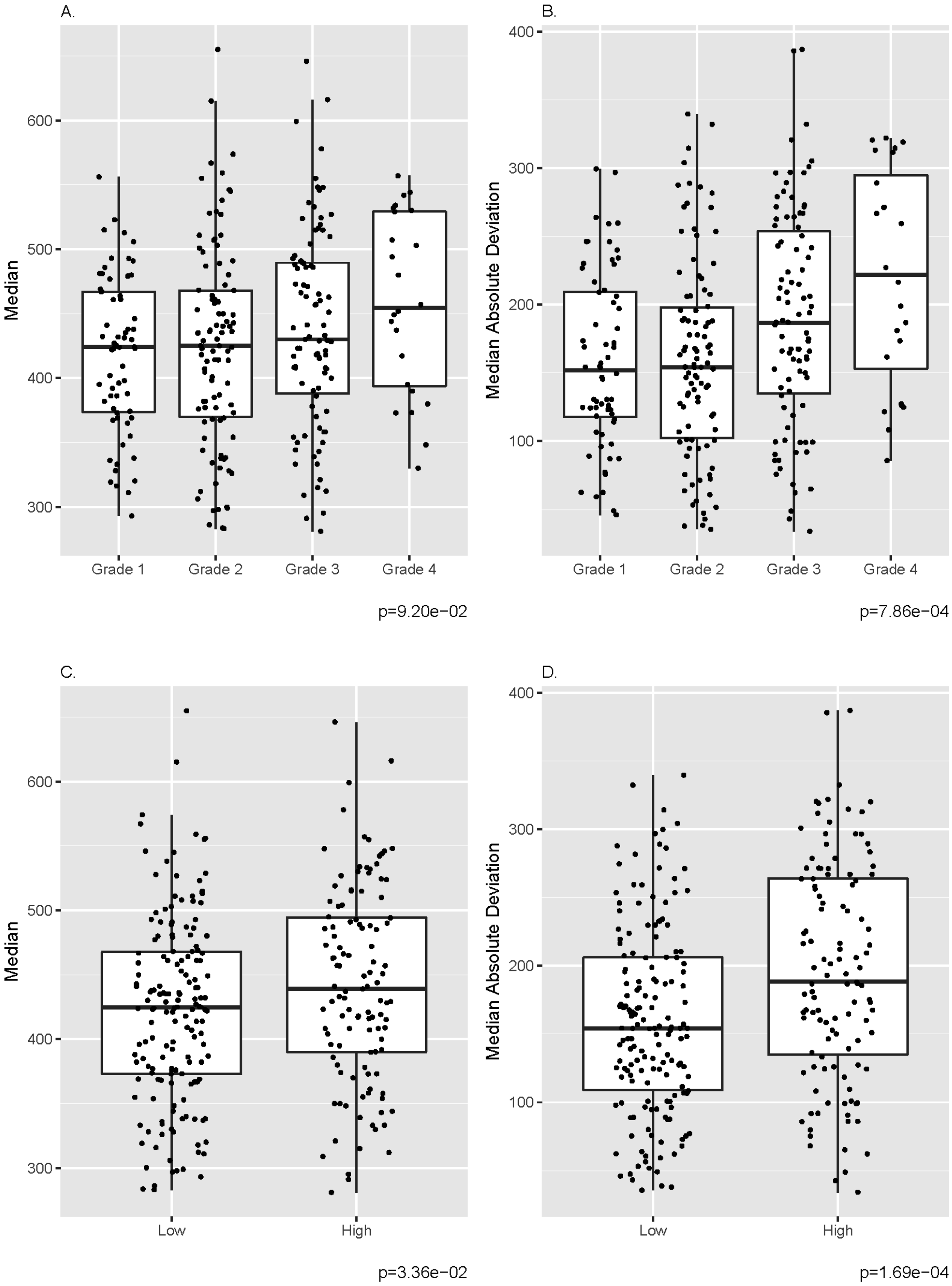

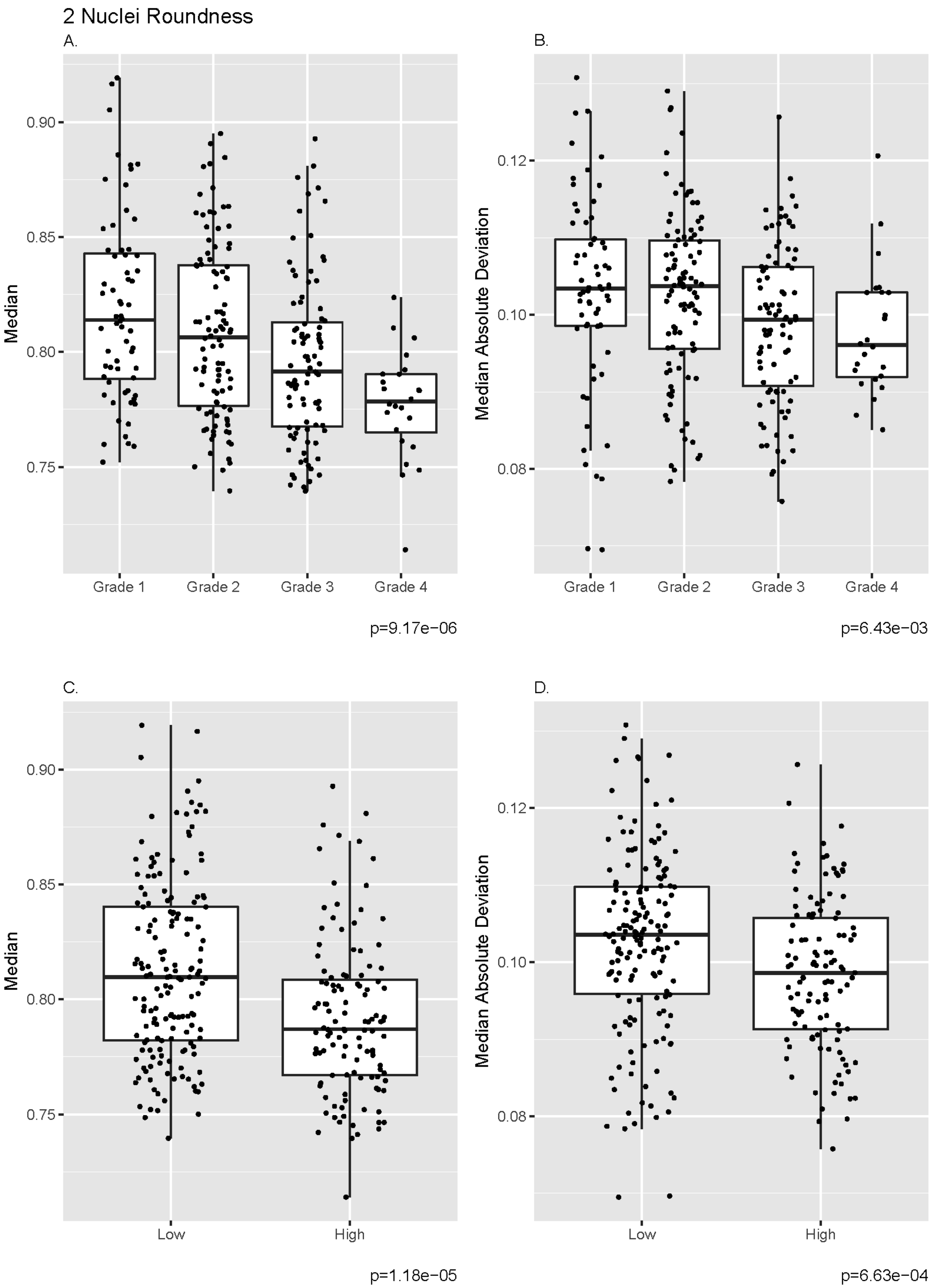
2 Nuclei Roundness
A.
Median
0.90
0.85
0.80
0.75
Grade 1
Grade 2
Grade 3
Grade 4
p=9.17e−06
B.
Median Absolute Deviation
0.12
0.10
0.08
Grade 1
Grade 2
Grade 3
Grade 4
p=6.43e−03
C.
Median
0.90
0.85
0.80
0.75
Low
High
p=1.18e−05
D.
Median Absolute Deviation
0.12
0.10
0.08
Low
High
p=6.63e−04

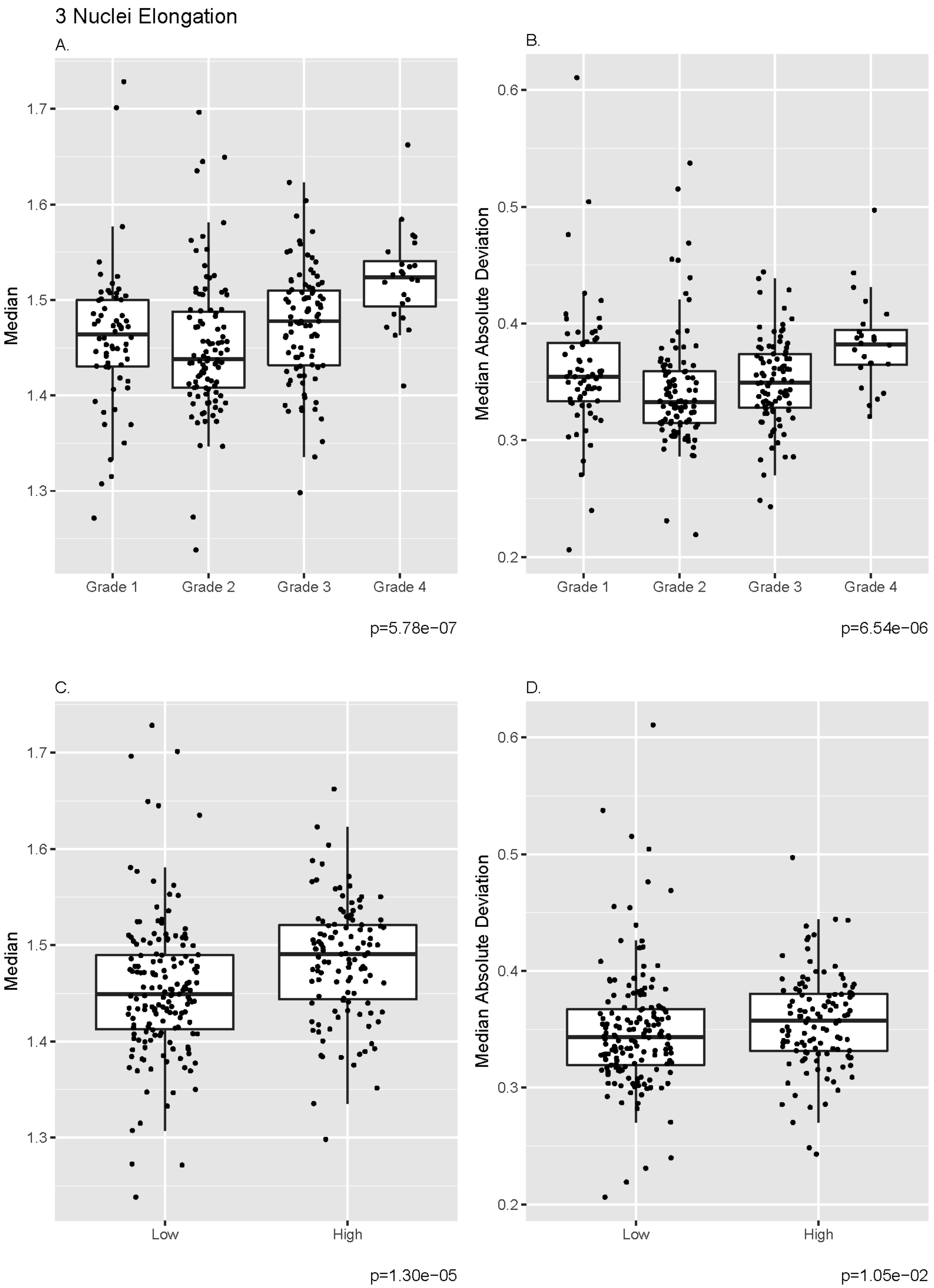
3 Nuclei Elongation
A.
Median
1.7
1.6
1.5
1.4
1.3
Grade 1
Grade 2
Grade 3
Grade 4
p=5.78e−07
B.
Median Absolute Deviation
0.6
0.5
0.4
0.3
0.2
Grade 1
Grade 2
Grade 3
Grade 4
p=6.54e−06
C.
Median
1.7
1.6
1.5
1.4
1.3
Low
High
p=1.30e−05
D.
Median Absolute Deviation
0.6
0.5
0.4
0.3
0.2
Low
High
p=1.05e−02

4 Nuclei Flatness

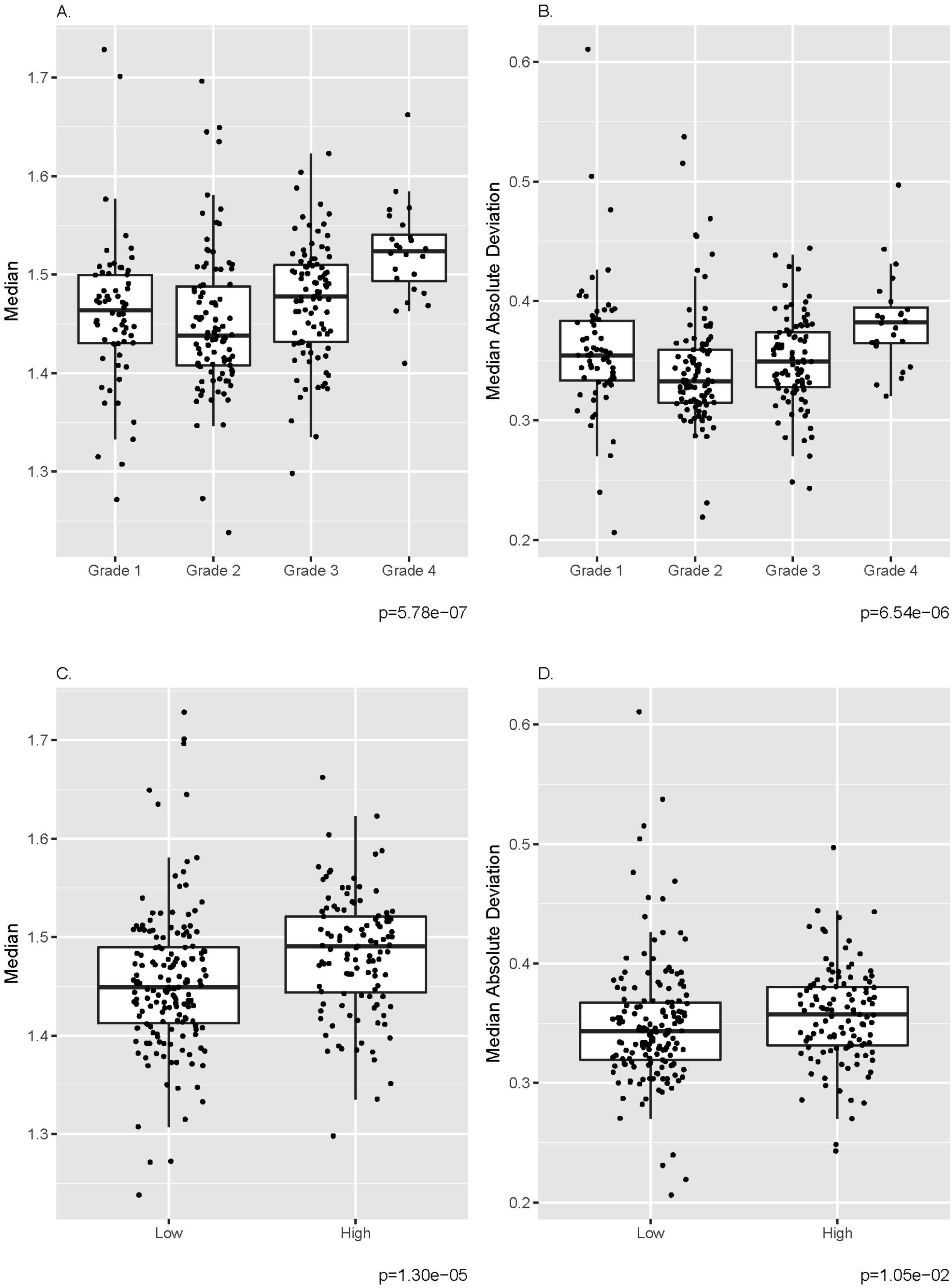

5 Nuclei Perimeter

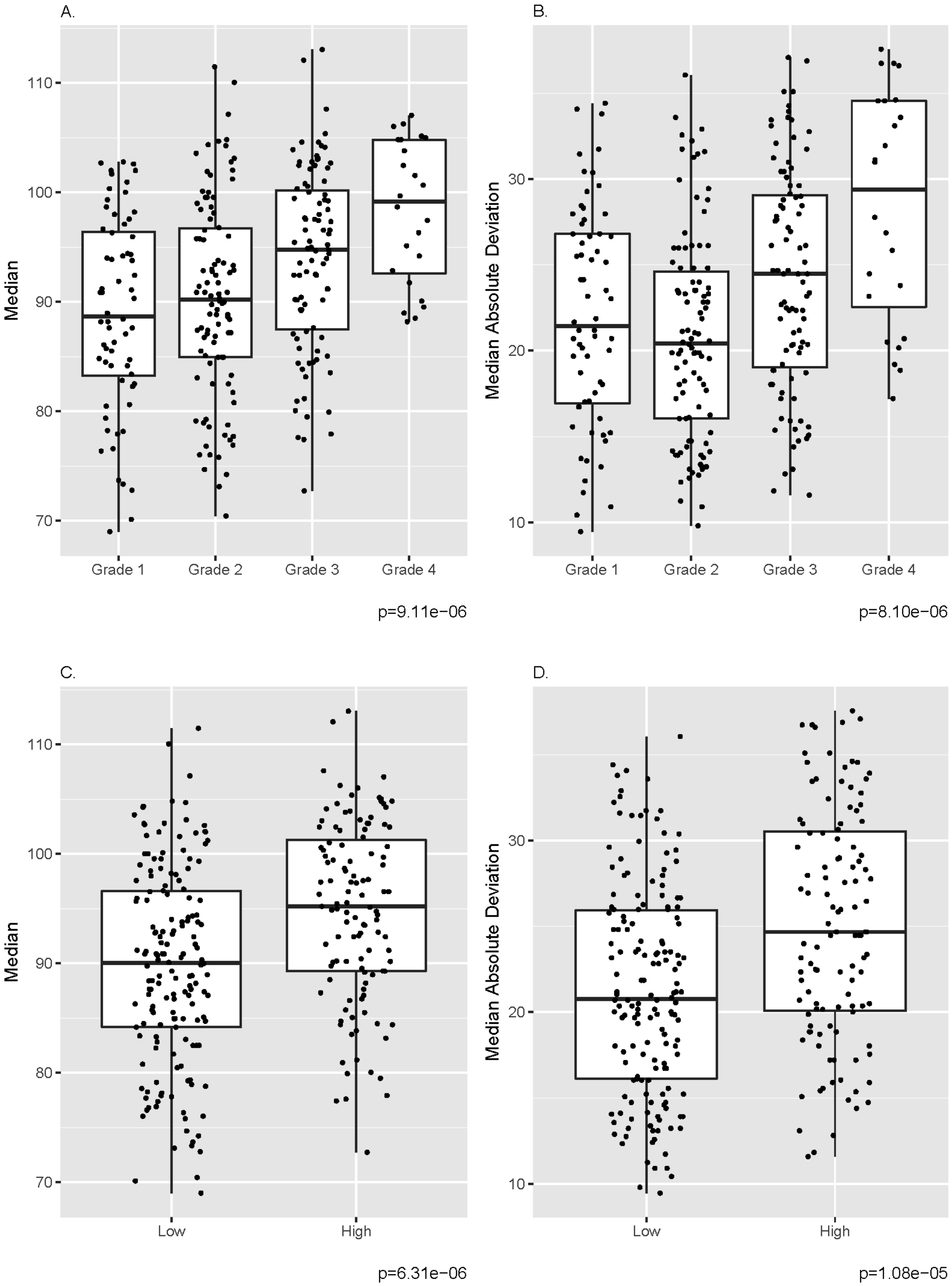

6 Nuclei Equivalent Spherical Perimeter

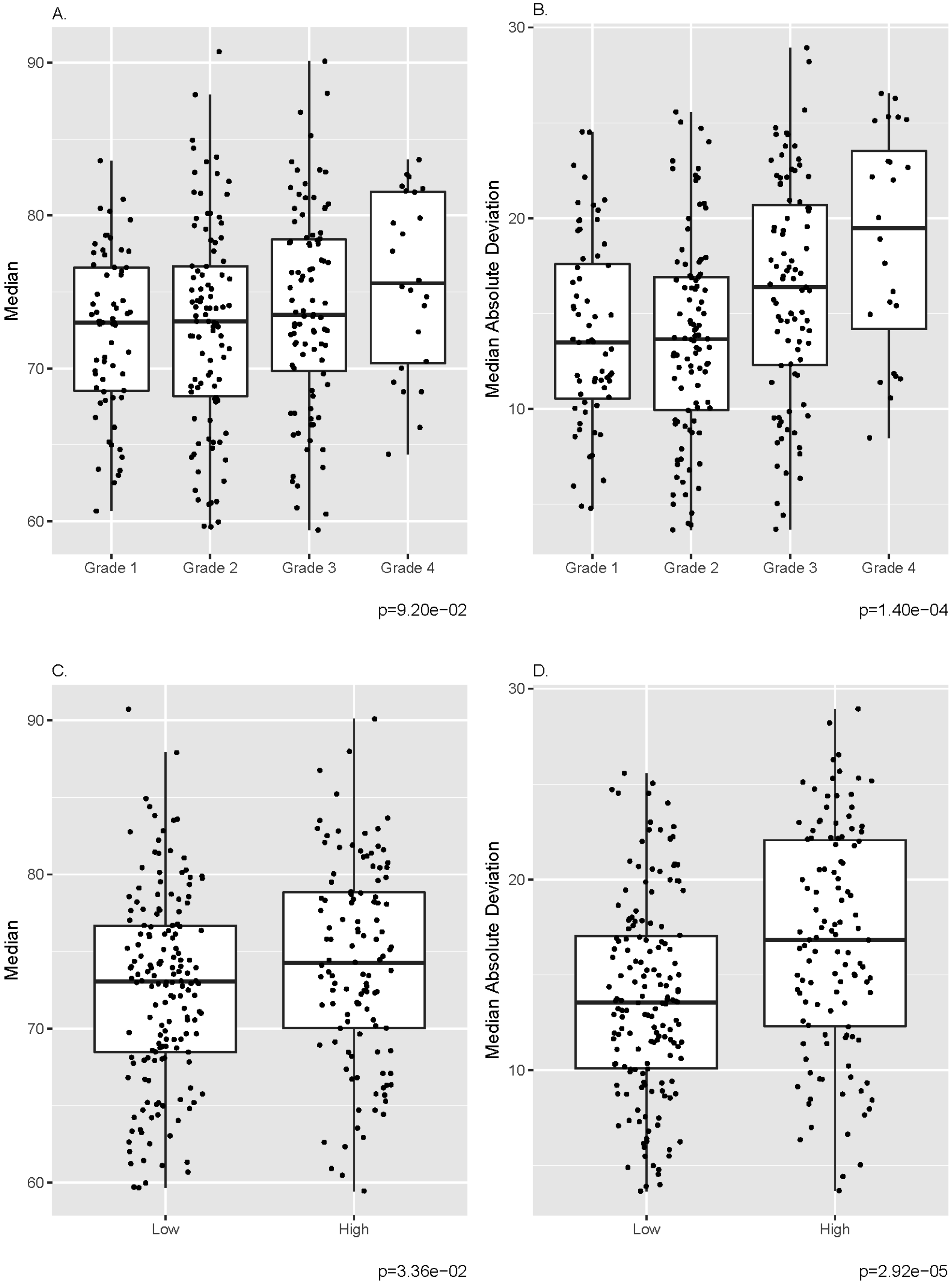

7 Nuclei Equivalent Spherical Radius

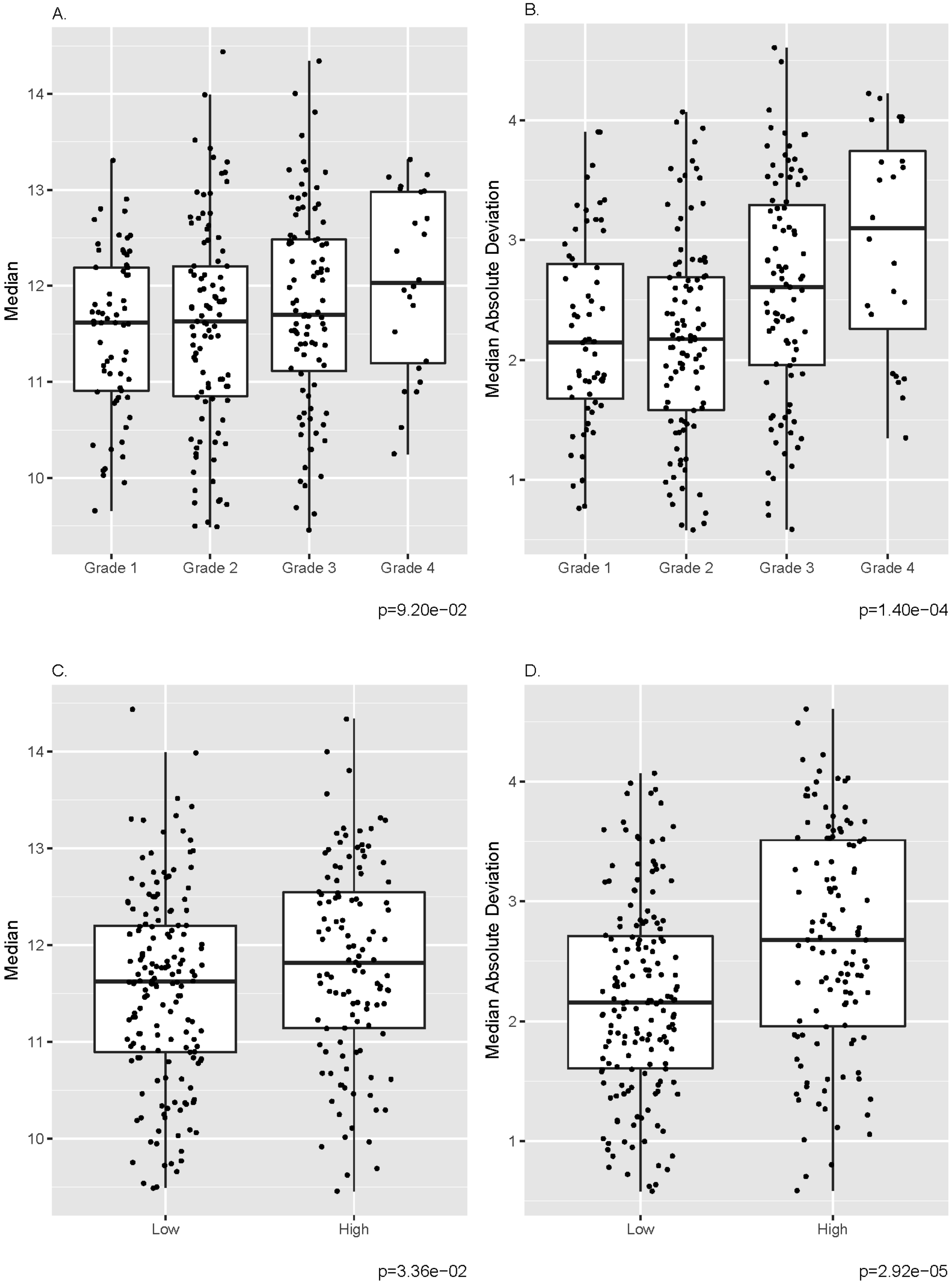

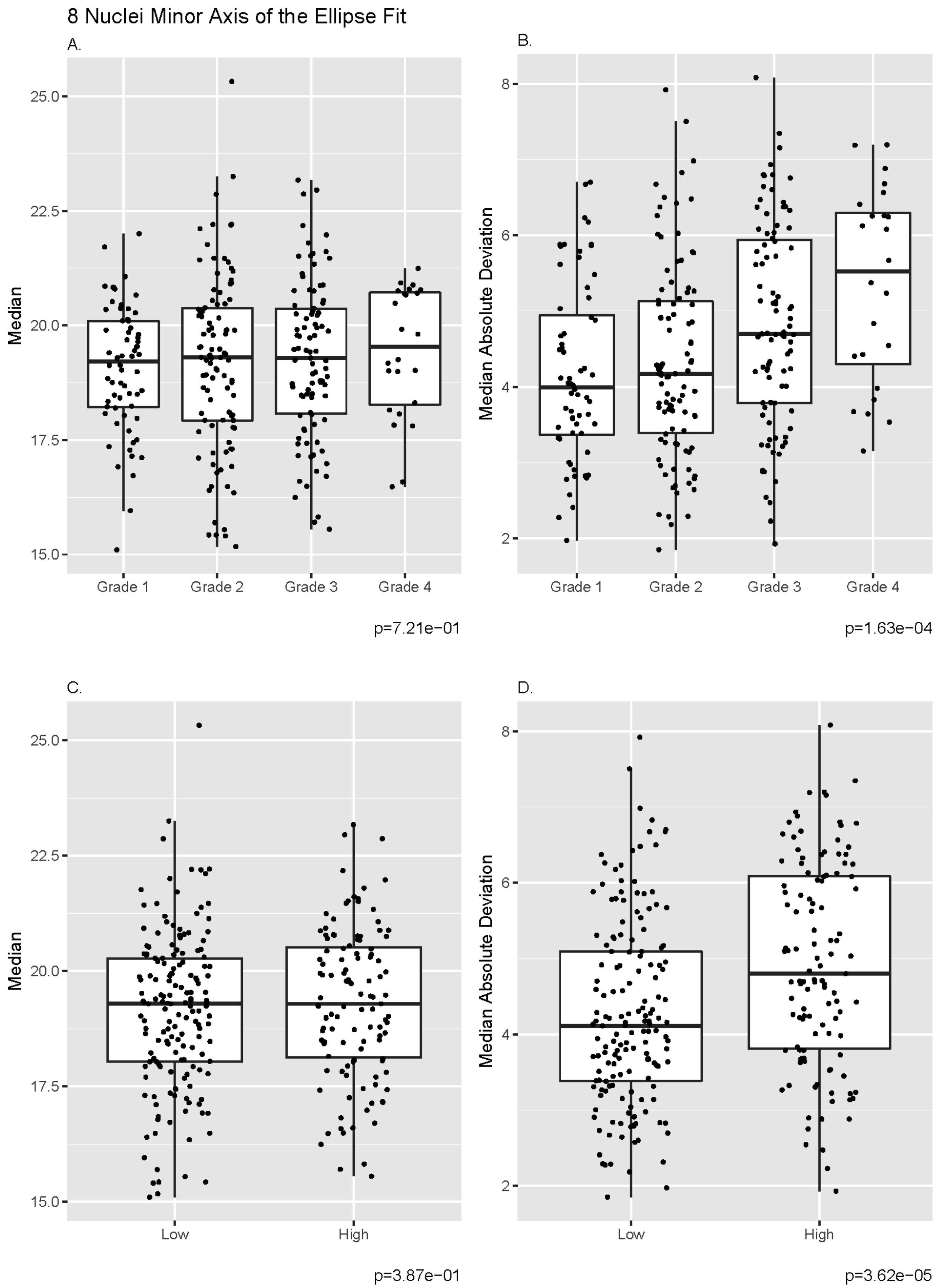
8 Nuclei Minor Axis of the Ellipse Fit
A.
B.
C.
D.
Median
Median Absolute Deviation
25.0
22.5
20.0
17.5
15.0
8
6
4
2
Grade 1
Grade 2
Grade 3
Grade 4
Low
High
p=7.21e−01
p=1.63e−04
p=3.87e−01
p=3.62e−05

9 Nuclei Major Axis of the Ellipse Fit

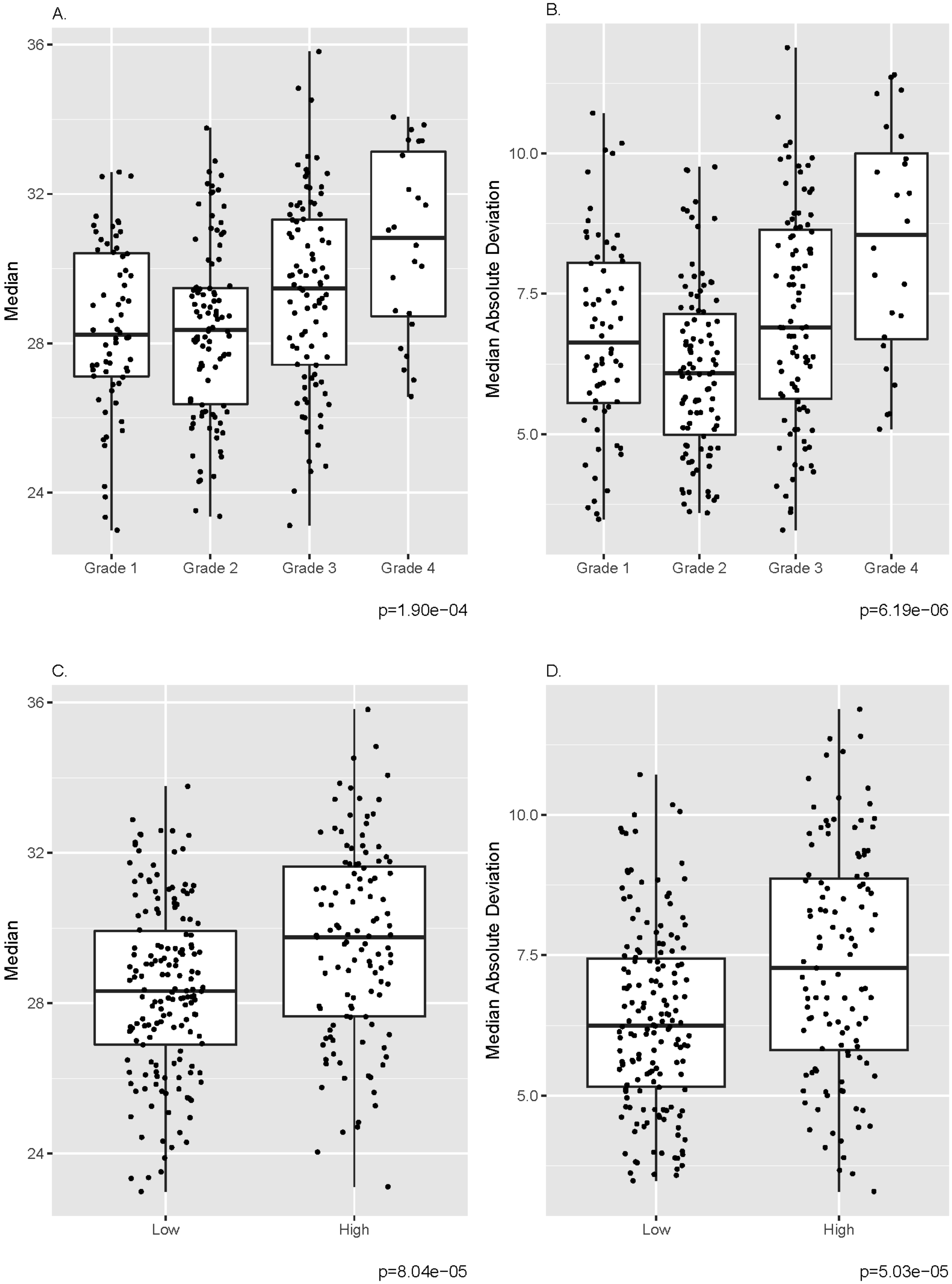

Supplement: S2 Fig — Nine morphological features (1: Area, 2: Roundness, 3: Elongation, 4: Flatness, 5: Perimeter, 6: Equivalent Spherical Perimeter, 7: Equivalent Spherical Radius, 8: Minor Axis of the Ellipse Fit, and 9: Nuclei Major Axis of the Ellipse Fit) were plotted with their median and median absolute deviation values. Each feature and measure was stratified either by the 4-tiered (A and B) or 2-tiered (C and D) grades assigned by Pathologist 1, respectively. (PDF) [file pone.0222641.s006.pdf]
